# Pilot study of repeated blood-brain barrier disruption in patients with mild Alzheimer’s disease with an implantable ultrasound device

**DOI:** 10.1186/s13195-022-00981-1

**Published:** 2022-03-08

**Authors:** Stéphane Epelbaum, Ninon Burgos, Michael Canney, Dawn Matthews, Marion Houot, Mathieu D. Santin, Carole Desseaux, Guillaume Bouchoux, Sebastian Stroer, Cyril Martin, Marie-Odile Habert, Marcel Levy, Aicha Bah, Karine Martin, Benoît Delatour, Maximilien Riche, Bruno Dubois, Lisa Belin, Alexandre Carpentier

**Affiliations:** 1 Sorbonne Université, Paris, France; 2 Institut du Cerveau – Paris Brain Institute – ICM, Paris, France; 3 Inserm, Paris, France; 4 CNRS, Paris, France; 5 Department of Neurology, Institute of Memory and Alzheimer’s Disease (IM2A), Pitié-Salpêtrière Hospital, AP-HP, Paris, France; 6 Pitié-Salpêtrière Hospital, AP-HP, Paris, France; 7 Aramis Project-Team, Inria-APHP Collaboration, Inria, Paris, France; 8 Carthera, Lyon, France; 9 ADM Diagnostics, Inc., Northbrook, IL USA; 10 Centre of Excellence of Neurodegenerative Disease (CoEN), Pitié-Salpêtrière Hospital, AP-HP, Paris, France; 11 Clinical Investigation Centre, Institut du Cerveau – Paris Brain Institute – ICM, Paris, France; 12 Center for NeuroImaging Research (CENIR), Institut du Cerveau – Paris Brain Institute – ICM, Paris, France; 13 Department of Neuroradiology, Pitié-Salpêtrière Hospital, AP-HP, Paris, France; 14 Department of Nuclear Medicine, Pitié-Salpêtrière Hospital, AP-HP, Paris, France; 15 Laboratoire d’Imagerie Biomédicale, Sorbonne Université, Inserm U 1146, CNRS UMR 7371, Pitié-Salpêtrière Hospital, Paris, France; 16 Centre Acquisition et Traitement des Images, Pitié-Salpêtrière Hospital, Paris, France; 17 Clinical Research Unit, Pitié-Salpêtrière Hospital, AP-HP, Paris, France; 18 Department of Biostatistics, Public Health and Medical Informatics, Pitié-Salpêtrière Hospital, AP-HP, Paris, France; 19 Department of Neurosurgery, Pitié-Salpêtrière Hospital, AP-HP, Paris, France

**Keywords:** Alzheimer’s disease, Clinical trial, Ultrasound, Florbetapir, Amyloid, Position emission tomography, Magnetic resonance imaging, Blood-brain barrier

## Abstract

**Background:**

Temporary disruption of the blood-brain barrier (BBB) using pulsed ultrasound leads to the clearance of both amyloid and tau from the brain, increased neurogenesis, and mitigation of cognitive decline in pre-clinical models of Alzheimer’s disease (AD) while also increasing BBB penetration of therapeutic antibodies. The goal of this pilot clinical trial was to investigate the safety and efficacy of this approach in patients with mild AD using an implantable ultrasound device.

**Methods:**

An implantable, 1-MHz ultrasound device (SonoCloud-1) was implanted under local anesthesia in the skull (extradural) of 10 mild AD patients to target the left supra-marginal gyrus. Over 3.5 months, seven ultrasound sessions in combination with intravenous infusion of microbubbles were performed twice per month to temporarily disrupt the BBB. ^18^F-florbetapir and ^18^F-fluorodeoxyglucose positron emission tomography (PET) imaging were performed on a combined PET/MRI scanner at inclusion and at 4 and 8 months after the initiation of sonications to monitor the brain metabolism and amyloid levels along with cognitive evaluations. The evolution of cognitive and neuroimaging features was compared to that of a matched sample of control participants taken from the Alzheimer’s Disease Neuroimaging Initiative (ADNI).

**Results:**

A total of 63 BBB opening procedures were performed in nine subjects. The procedure was well-tolerated. A non-significant decrease in amyloid accumulation at 4 months of − 6.6% (SD = 7.2%) on ^18^F-florbetapir PET imaging in the sonicated gray matter targeted by the ultrasound transducer was observed compared to baseline in six subjects that completed treatments and who had evaluable imaging scans. No differences in the longitudinal change in the glucose metabolism were observed compared to the neighboring or contralateral regions or to the change observed in the same region in ADNI participants. No significant effect on cognition evolution was observed in comparison with the ADNI participants as expected due to the small sample size and duration of the trial.

**Conclusions:**

These results demonstrate the safety of ultrasound-based BBB disruption and the potential of this technology to be used as a therapy for AD patients. Research of this technique in a larger clinical trial with a device designed to sonicate larger volumes of tissue and in combination with disease-modifying drugs may further enhance the effects observed.

**Trial registration:**

ClinicalTrials.gov, NCT03119961

**Supplementary Information:**

The online version contains supplementary material available at 10.1186/s13195-022-00981-1.

## Background

Alzheimer’s disease (AD) is a growing global health concern with an annual incidence of 1.8 million people in the USA and Europe [1]. Although an understanding of the underlying pathophysiology of the disease has grown over the past several decades, no effective treatments exist that slow cognitive decline.

AD is characterized by an accumulation of β-amyloid in plaques and neurofibrillary tangles composed of tau in the brain [2–6]. Both β-amyloid and tau have been the targets of extensive drug development [1], with aducanumab, a human monoclonal antibody that selectively binds to β-amyloid fibrils and soluble oligomers, provisionally approved by the Food and Drug Administration in the USA in 2021 [7, 8]. However, such treatments’ effect on amyloid load and cognitive function are dose-dependent [9], and only 0.1% of intravenously injected anti-Aß immunoglobulins reach the brain, despite a half-life of 15–20 days [10].

The poor penetration of current antibody therapies for AD is due to their large size (> 150 kDa) and the presence of the blood-brain barrier (BBB), which limits 98% of small (< 500 Da) and almost 100% of larger (> 500 Da) molecules from entering the brain parenchyma [11]. Thus, there is a need to improve the bioavailability of these antibodies in the brain to improve their efficacy.

The use of low-intensity pulsed ultrasound in combination with systemic injection of microbubbles has been explored for the past two decades as a method to temporarily disrupt the BBB [12]. Ultrasound-based blood-brain barrier disruption (US-BBBD) allows for increased penetration of systemically administered small and large molecule drug therapies into the brain [13–15]. Strikingly, US-BBBD alone has been shown to reduce β-amyloid and tau pathologies, stimulate neurogenesis, and improve cognitive performance in mouse AD models [16–18] and can be further coupled with drug therapies to improve their brain penetration and efficacy [13, 19, 20].

Clinical-stage devices have been developed using extracranial [21] and implantable [22, 23] approaches. These devices are being tested in clinical trials in patients with brain tumors [23, 24] and neurodegenerative diseases [25, 26]. Recently, the safe disruption of the BBB in six AD patients using a transcranial focused ultrasound system was demonstrated [27, 28].

The goal of this phase 1/2 clinical trial was to test the hypothesis that US-BBBD using an implantable ultrasound device is safe in early AD patients and could lead to a reduction in β-amyloid pathology when used alone. Seven repeated sonications every 2 weeks were performed during the first 4 months after study inclusion to disrupt the BBB. Positron emission tomography (PET), magnetic resonance imaging (MRI), and cognitive assessments were used to monitor treatments and disease progression at 0, 4, and 8 months after inclusion.

## Methods

### Study design

This investigator-sponsored phase I/II trial was a single-center, exploratory clinical trial (NCT03119961) initiated at Hôpital de la Pitié Salpêtrière (Paris, France). The primary objective was to evaluate the changes on PET imaging on β-amyloid and glucose in the region of interest (ROI) targeted by the ultrasound device. Secondary objectives were to assess the radiographic and clinical tolerance of repeated BBB opening by ultrasound, to examine the opening of the BBB on T1-weighted (T1w) MRI, and to study the evolution of cognitive decline. The study was approved by the Paris VI Ethical Committee. Informed consent was obtained from all participants.

Patients between the ages of 50–85, with early-stage AD (Mini-Mental State Examination [MMSE] 20–26) were eligible. Inclusion was based on cognitive assessment [29] and an MRI showing one of the three most frequent phenotypic presentations of the disease (hippocampal amnesia, logopenic aphasia, or posterior cortical atrophy syndrome). The diagnosis was confirmed by the presence of cerebrospinal fluid levels of ptau/Aβ1–42 > 0.11 [30].

No control subjects were included in the study, but 45 controls were sampled from the Alzheimer’s Disease Neuroimaging Initiative (ADNI) database (adni.loni.usc.edu) through a matching procedure taking into account age, gender, MMSE score, and diagnosis (mild cognitive impairment/AD).

### Ultrasound device

**Fig. 1:**
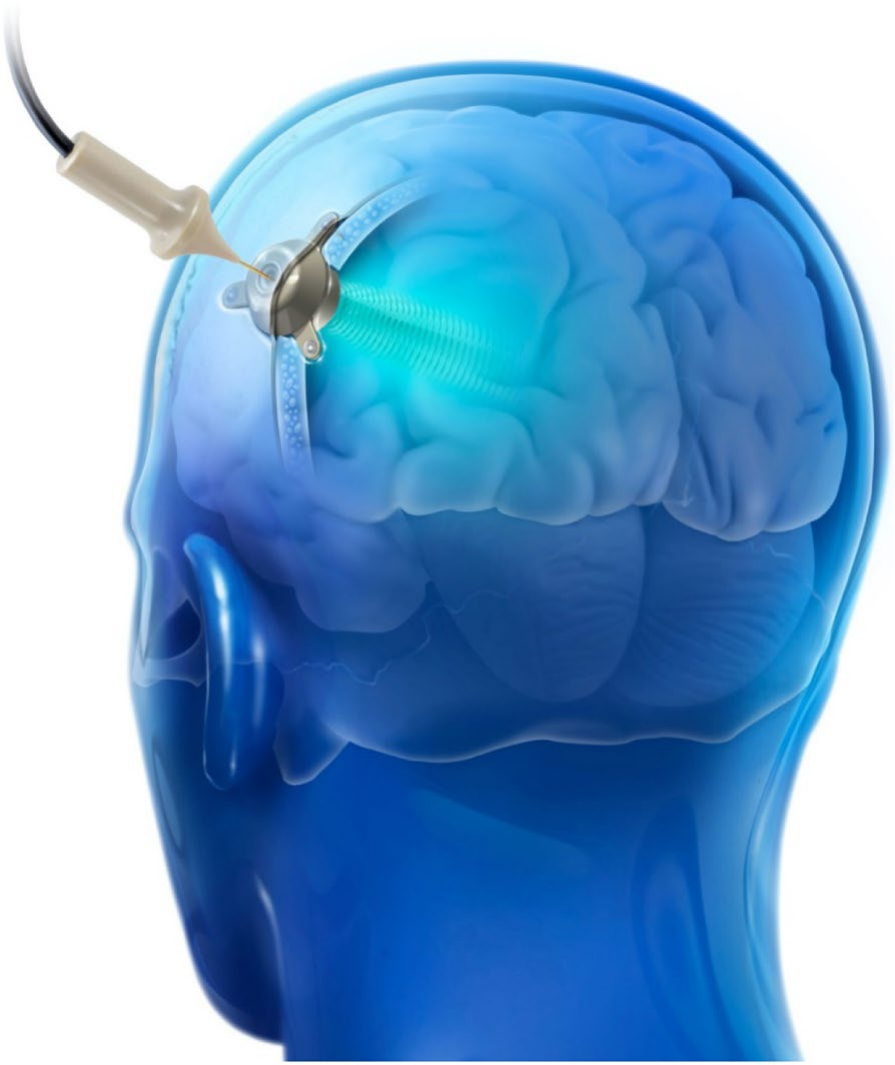
The SonoCloud-1 is a 1-MHz implantable ultrasound device that is implanted within the skull thickness (extradural) and activated at each treatment cycle by connecting it using a transdermal needle to an external radiofrequency generator. In this study, the SonoCloud-1 was implanted to target and temporarily disrupt the BBB above the left supramarginal gyrus

The SonoCloud-1 implantable ultrasound device (Carthera, Paris, France) was used for sonications (Fig. 1). This investigational device was previously used in a phase 1/2a study in patients with recurrent glioblastoma who had monthly repeated ultrasound-mediated BBB opening prior to receiving carboplatin chemotherapy [22, 23]. The device consisted of a 10-mm diameter, 1-MHz ultrasound transducer encapsulated in a biocompatible housing. The acoustic pressure field of the device is shown in Additional file 1: Fig. S4 and described further in Asquier et al. [31]. The device was placed in a 12-mm diameter burr hole in the left parietotemporal junction targeting the left supramarginal gyrus using a neuronavigation system under local anesthesia. To activate the device, it was connected using a transdermal needle to a radiofrequency generator, with the first activation occurring at least 15 days after device implantation. During sonications, a 25,000-cycle pulse was used every second for a duration of 4 min in combination with intravenous injection of SonoVue® microbubbles (0.1 mL/kg, Bracco). The device was activated every 2 weeks over the course of seven sessions after patient inclusion (3.5 months). The acoustic pressure, initially set at 0.9 MPa, was increased after the first sonication session to 1.03 MPa. This pressure range was selected as it allowed for reproducible safe and efficient BBB disruption in glioblastoma patients during a prior study [13]. At 9 months after implantation, the device was explanted.

### Safety assessments

Safety assessments included physical and neurologic examinations and the collection of adverse event (AE) data according to the Common Terminology Criteria for Adverse Events (CTCAE) v4.0.

### Cognitive evaluation

The neuropsychological evaluations were performed at baseline, 4 months, and 8 months. The evaluation comprised the MMSE [32], Clinical Dementia Rating Scale Sum of Boxes Score (CDR-SB) [33, 34], Frontal Assessment Battery (FAB) [35], the Free and Cued Selective Reminding Test (FCSRT) [36], Trail Making Test (TMT) [37], verbal fluencies [38], praxis [39], Rey’s figure [40], State-Trait Anxiety Inventory: STAI (Form Y) [41], and Montgomery-Åsberg Depression Rating Scale (MADRS) [42].

### MRI/PET imaging acquisition

MRI imaging was performed following the BBB opening procedure during the first and third sessions on a 3-T Prisma Fit (Siemens, Erlangen, Germany) using a 64-channel head coil for signal reception. T2-FLAIR-weighted images (1 mm isovoxel) and diffusion-weighted images (2 mm isovoxel) were acquired for monitoring for any potential edema induced by the BBB disruption procedure. Quantitative susceptibility mapping images (1-mm isovoxel) were obtained using multi-echo T2*-weighted images to detect any potential hemorrhages. To evaluate BBB disruption, T1 maps (1-mm isovoxel) were then obtained with the MP2RAGE sequence before and 7 min after a bolus injection of 0.2 mL/kg gadolinium-based contrast agent (Gd-DOTA, DOTAREM, Guerbet, France). These images were planned to be performed 60 min after sonication.

PET imaging to examine amyloid and glucose in the brain was performed at 0, 4, and 8 months after subject inclusion. PET acquisitions were performed on the PET/MR SIGNA 3T system (GE Healthcare) after the implantation of the device. The two acquisitions took place 48 h apart. Amyloid PET imaging started 50 min after intravenous injection of 370 MBq of ^18^F-florbetapir, and FDG PET imaging started 30 min after intravenous injection of 2 MBq/kg of ^18^F-fluorodeoxyglucose (FDG). During the period of FDG tracer uptake, participants were at rest with eyes open but ears closed to minimize MRI scanner noise. The 50-min post-injection start time for amyloid PET was used to maximize a pseudo-equilibrium state. For both radiotracers, acquisition parameters were as follows: simultaneous PET/MRI acquisition with (i) 20-min PET acquisition; (ii) acquisition of four T1 DIXON sequences: in-phase, opposed-phase, fat-only, and water-only, and a zero echo time sequence to capture bone information [43]; the five images are combined to create a μ map, used for attenuation correction of the images; and (iii) 3D T1w anatomical sequence.

### BBB disruption efficiency

To evaluate BBB disruption efficacy, the map of Gd-DOTA concentration was calculated from the difference of the registered T1 maps, considering a T1 relaxivity of 4.5 mM^−1^ s^−1^ [44]. As a metric for BBB disruption efficacy, the total quantity of Gd-DOTA in sonicated brain tissues was calculated in a 15 × 55 mm cylindrical ROI covering the ultrasound beam generated by the implant and compared with the Gd-DOTA quantity in a symmetric contralateral control ROI (Additional file 1: Fig. S3). The volume of brain voxels with an enhanced concentration of Gd-DOTA was also calculated in the ROI, using a concentration threshold automatically adjusted such that less than 5% of the control ROI was classified as enhanced. An ultrasound-mediated BBB opening was considered successful if the quantity of Gd-DOTA in the ROI was greater than the quantity in the symmetric control ROI plus two standard deviations of all control ROIs. BBB opening was also visually assessed as in our previous study [22].

### Image processing

Scans (time frames already averaged for PET) were visually inspected for anatomical completeness, subject motion, and other artifacts and converted to NIFTI format. MRI scans (0.488 × 0.488 × 1.2 mm) were resliced to 1 × 1 × 1 mm and processed using CorInsights MRI, which uses Freesurfer 6.0 and other algorithms for segmentation. PET scans were co-registered to their corresponding resliced volumetric MRI scans as produced by Freesurfer. PET scans obtained at 4 and 8 months were additionally coregistered to their corresponding initial scans. Baseline MRI scans were spatially transformed to template space using SPM12, and the transformation was applied to the co-registered PET scans.

### Amyloid PET analysis

To confirm the presence of amyloid at baseline and to assess longitudinal changes in the brain regions that are typically amyloid positive in Alzheimer’s disease, values were measured in the posterior cingulate, precuneus, lateral temporal, frontal, and anterior cingulate regions. SUVRs were evaluated using the whole cerebellum and eroded subcortical white matter as comparative reference regions. SUVRs in the global cortex and a relatively large temporoparietal region were also measured using additional processing and reference region approaches as described below. A visual read was also performed at baseline.

**Fig. 2:**
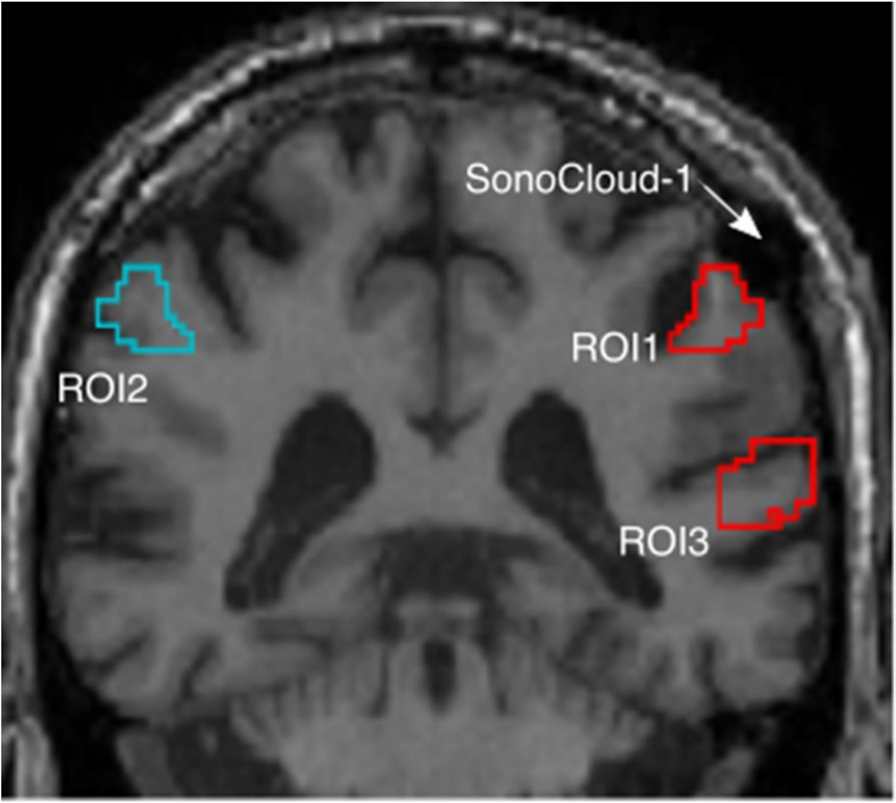
Region of interest (ROI) definition to measure the changes in PET amyloid levels in gray matter targeted by the implant (ROI1) as compared to similar tissue in the opposite (ROI2) and same hemispheres (ROI3)

To evaluate the local sonication effects, a custom volume of interest was created for each participant centered at the implant location and extending inward approximately perpendicular to the skull at the position of the implant, with initial dimensions of 10 × 10 × 40 mm^3^. A thresholded version of each volume was created to eliminate the cerebrospinal fluid (CSF) from the measured boundaries. Additional custom volumes of interest were created to measure distal tissue in the same coronal slices within the same hemisphere, as well as in the opposite hemisphere, serving as comparator ROIs (Fig. 2). The ROIs local to the implant were additionally restricted to include only gray matter to assess the effects of including white matter (which provided a slightly larger ROI less vulnerable to technical or motion-related variability) upon measured values.

Standardized uptake value ratios (SUVRs) were calculated as the ratio of the value in the implant ROI divided by the value in each of the comparator ROIs as the reference (same hemisphere and opposite hemisphere, separately). This approach minimized the technical variability that can arise from using a reference region located in distant slices of the brain, while maximizing similarities in tissue kinetics. SUVRs were also calculated relative to the overall bilateral parietal region. This provided a comparison to similar tissue at a similar general spatial location within the brain but with a larger volume to reduce technical noise. The whole cerebellum and white matter were also evaluated as reference regions, but this was for information only given technical noise associated with the cerebellar reference in longitudinal measurement [45] and the better spatial and tissue type match obtained using adjacent and opposite hemisphere tissue as the reference.

The reliability of the target region amyloid measures for each subject was assessed by determining whether unacceptable embedded head motion had occurred during the scan. Motion would be indicated by a spiral artifact in the MRI scan (acquired in the same session and position as the PET scan) and/or by a longitudinal change in the regions distant from the implant that were well beyond the range expected over 4 and 8 months physiologically based upon numerous studies [45].

### FDG PET analysis

FDG PET scans were evaluated using the same standard and custom ROIs and reference region approaches that were defined for the analysis of the amyloid scans. In addition, a voxel-based multivariate machine learning classification software was applied to explore the patterns discriminating baseline, month 4 (M4), and month 8 (M8) states. Briefly, the spatially normalized, smoothed FDG scans for the three time points were grouped into three (*N*) training classes. Using the NPAIRS software framework, principal components (PCs) were determined for the data set, after which canonical variate analysis (CVA, a form of linear discriminant analysis) was used to mathematically combine the most significant PCs into *N*-1 (two) patterns of hypometabolism and hypermetabolism or preservation relative to the whole brain. The data set was split into halves numerous times, each time using each half to generate a model (patterns) and generating a reproducibility metric as the correlation between the patterns, and a prediction metric based on the classification of one half from the other half’s model [46]. Consensus patterns were derived based upon these metrics, and the scores for the primary pattern (CV1) were compared across the groups and individuals.

### Global-scale analyses and comparison with an external control cohort

Potential effects upon amyloid and FDG PET were additionally evaluated by comparing SUVRs for the whole cortex and a temporoparietal ROI (comprising the angular, supramarginal, and superior temporal gyri) in the hemisphere of the implant versus the opposite hemisphere and a slightly different set of processing steps described in supplementary materials. This provided a comparator to the standard ROI analyses that had been performed using somewhat different technical approaches and reference regions.

As no control group was recruited in the study, we selected patients from the ADNI database [47]. The selection was made from ADNI subjects who had at least two sessions (on average 30 months apart) with T1w MRI, FDG, and amyloid PET data and with an MCI or AD diagnosis at the considered sessions [48, 49]. For each subject, the five ADNI subjects closest in terms of age, with the same gender and no more than a 2-point difference in MMSE, were selected. The images were processed using the same procedure as the one used for the study subjects but restricted to two time points.

### Statistical analysis

Evolution in regional PET SUVR was tested between M0 and M8 for both the FDG and amyloid tracers using the Wilcoxon signed-rank test. Evolution in neuropsychological scores was compared between M0 and M4, between M4 and M8, and between M0 and M8, using the Wilcoxon signed-rank tests. To correct for multiple testing, we used the Benjamini-Hochberg method. We compared our population to the control ADNI population in terms of demographic characteristics such as age and MMSE and in terms of regional PET SUVR computed at baseline in both the large and small ROIs using the Kruskal-Wallis *H*-test. We also compared the annualized percent change in cognitive and PET SUVR between these two groups. The Benjamini-Hochberg method was also used to correct for multiple testing. All patients with at least one sonication performed were analyzed for efficacy. Safety was described on all included patients.

## Results

**Fig. 3:**
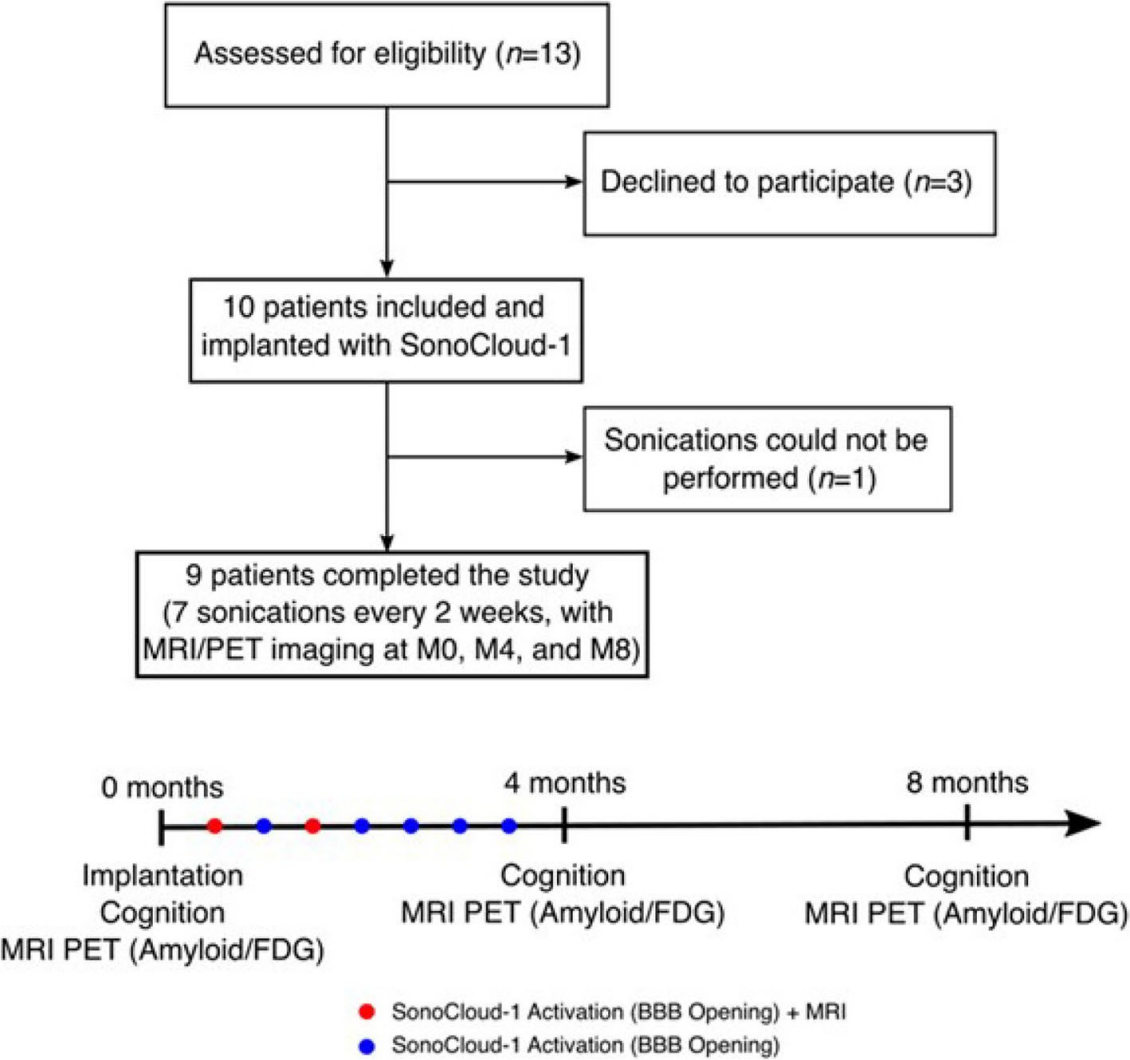
Trial overview

An overview of the trial is shown in Fig. 3. Thirteen patients were screened, ten were included and implanted, and nine patients completed the trial. The demographics of these patients are shown in Table 1. The patients had mild AD, with a median age of 71 years, 14 years of education, and a MMSE of 25.

**Table 1:** Patient demographics

	Patients analyzed (*N* = 9)
Gender (*female*)	5 (55.6%)
Age (*years*)	71.0 [69.0, 73.0]
Education (*years*)	14.0 [12.0, 15.0]
MMS	25.0 [21.0, 26.0]
FR	4.5 [4.0, 6.0]
TR	11.5 [8.75, 18.75]
intr	18.0 [7.0, 28.0]
Rey copy	29 [20.0, 32.0]
Prax	20.0 [17.0, 21.0]
Rey mem	4 [2, 6]
FAB	14.0 [13.0, 17.0]
DelFR	2.0 [0.0, 3.0]
DelTR	4.0 [0.0, 11.0]
Cat fluency	17.0 [15.0, 24.0]
Lit fluency	16.0 [13.0, 25.0]
STAI	49 [39, 55]
MADRS	5.0 [5.0, 9.0]
CDR-SB	3.5 [2.5, 4.0]
TMT B-A_time	88.0 [61.0, 167.5]

Data are given as median [first quartile, third quartile] for continuous variables and as count (percentages) for categorical variables. *MMS* Mini-Mental State, *FR* free recall, *TR* total recall, *intr* intrusions, *Prax* praxis, *FAB* frontal assessment battery, *DelFR* delayed free recall, *DelTR* delayed total recall, *Cat* categorical, *Lit* literal, *STAI* State-Trait Anxiety Inventory, *MADRS* Montgomery-Åsberg Depression Rating Scale, *CDR-SB* Clinical Dementia Rating Scale Sum of Boxes, *TMT* Trail Making Test

The treated patients received a total of seven sonications each (every 2 weeks) for a total of 63 sessions to disrupt the BBB using the SonoCloud-1 including nine sessions (1st sonication in each patient) at 0.9 MPa and 54 sessions at 1.03 MPa. The procedure was well-tolerated at both pressure levels used. No patient had redness on the implantation area or pain, no skin infection, and no systemic infectious symptom. The time to connection of 4.53 min on average (± 9.29 min) and a median pain evaluation during needle connection of 2 on a scale of 10 were observed in patients.

### Safety and feasibility

One patient was prematurely withdrawn from the study after no connection was established after two attempts. An MRI revealed a particularly thick scalp in this participant (8 mm), which led to the connection difficulty and led to revised non-inclusion criteria to prevent such difficulties in the future.

A total of thirteen AEs (detailed in Table 2) were reported, with all except one event a grade 1. One severe adverse event occurred during the trial. The fourth participant suffered from delirium for 2 h. This event occurred 2 days after the second BBB opening session. A brain MRI revealed bleeding on a previously existing microbleed in the left frontal hemisphere, which was 5 cm from the ultrasound sonication region. An Independent Data Safety Monitoring Board (DSMB) concluded there was an unlikely relation of this AE to the BBB opening procedure, and the participant agreed to continue sonications on the protocol. No subsequent AEs occurred for this participant after this event even after sonications were resumed.

**Table 2:** Adverse events description. Treatment-emergent adverse events (CTCAE version number) which occurred during treatment or up to 30 days after the end of therapy. The occurrence of each adverse event is listed as well as the total number of patients affected as some patients might have experienced the same adverse event multiple times over the course of therapy

*N* = 10 patients	Number of events by grade		Number (percentage) of participants
	Grade 1	Grade 2	
Delirium with frontal microbleed		1	1 (10%)
Fatigue	2		2 (20%)
Back pain	1		1 (10%)
Dandruff	1		1 (10%)
Prurit	1		1 (10%)
Asymptomatic low blood pressure	1		1 (10%)
Right hand sensitive deficit	1		1 (10%)
Headaches (intensity range on a visual scale, 2–4/10)	2		2 (20%)
Diarrhea	1		1 (10%)
Abdominal pain	2		1 (20%)

Transdermal needle/implant connection issues were reported for 11 sessions, but none of them had any consequences for the patient (no pain or AE). In the sessions including an MRI after BBB opening, no immediate AEs were detected radiologically, with no changes in FLAIR or T2* and diffusion-weighted imaging.

### Amyloid PET results

Each of the nine participants who completed the study had a positive amyloid burden at baseline based upon the visual read of their florbetapir PET scan as well as the measurement of regional SUVRs (Additional file 1: Fig. S1). Within the tissue proximal to the SonoCloud-1 implant, 8/9 participants had a positive amyloid burden (AV-45 SUVR referenced to the whole cerebellum below 1.1), with patient 10 at the limit of positivity (value = 1.11).

Two participants who were amyloid-positive in the target region showed evidence of technical artifacts making measurement uncertain. In one participant, the baseline MRI scan had a prominent ring artifact characteristic of significant head motion. For these participants, SUVRs in regions unlikely to be affected by sonication exhibited longitudinal change outside of an expected physiologic range. These two subjects were excluded from the result graphs from the calculation of amyloid changes in the target region described below.

**Fig. 4:**
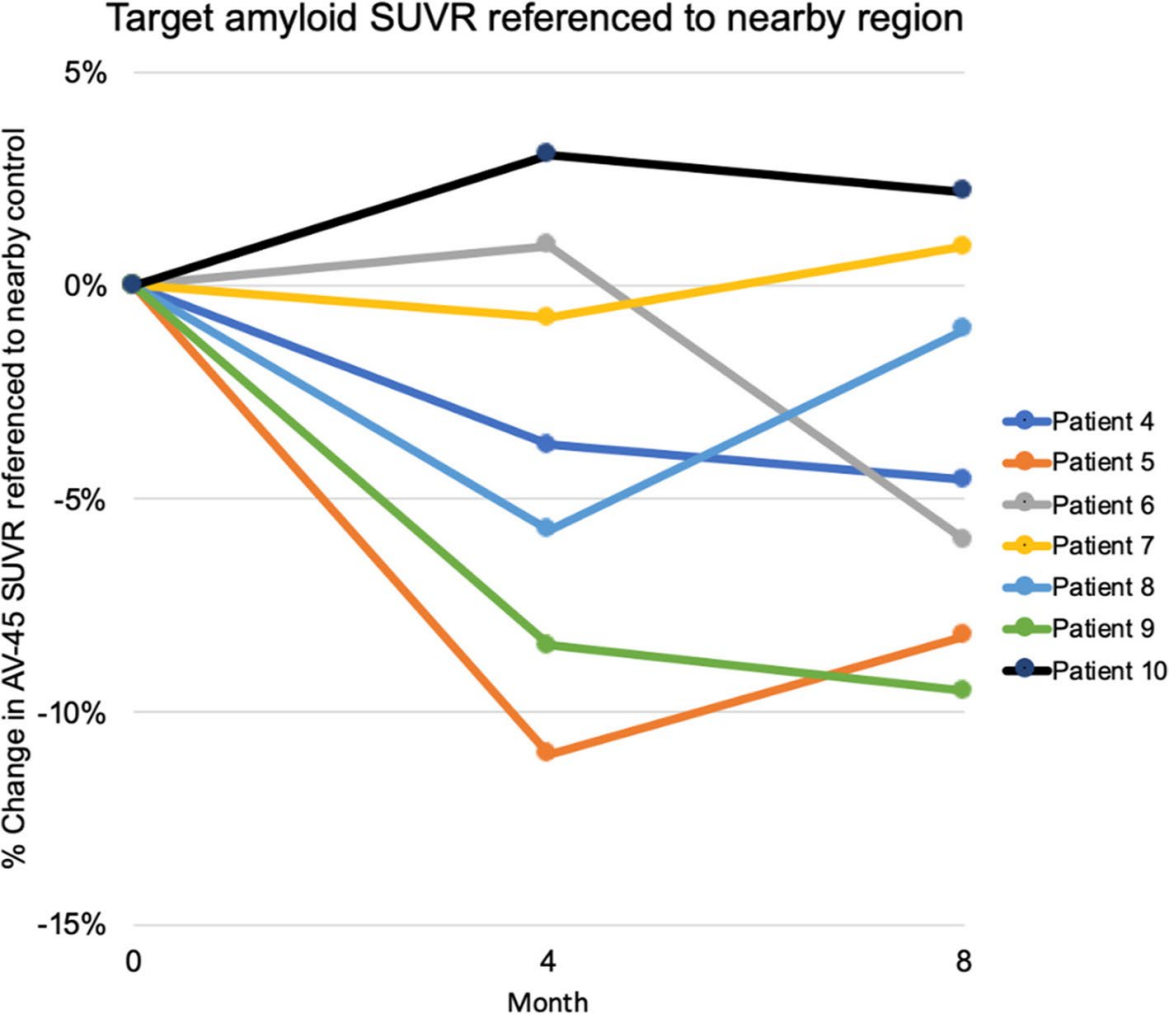
PET amyloid (florbetapir) change observed in the region targeted by the SonoCloud-1 device (ROI1). SUVRs referenced to a ROI similar to the target ROI but in the opposite hemisphere (ROI2) showed decreases from the baseline of − 6.6% (SD = 7.2%) at 4 months and − 5.7% (SD = 6.2%) at 8 months

SUVRs referenced to a ROI similar to the target ROI but in the opposite hemisphere showed decreases in amyloid in participants with a positive baseline amyloid value and passing quality control. The mean and (SD) change from baseline for these seven participants was − 0.73 (0.081) and − 0.64 (0.064) for 4 and 8 months post-baseline, respectively, or − 6.6% (7.2%) and − 5.7% (6.2%) on a percentage basis (Fig. 4). Using a similarly sized ROI in the same hemisphere, the mean changes in these participants for 4 and 8 months were − 0.05 (0.049) and − 0.049 (0.044) for 4 and 8 months, or − 4.8% (4.5%) and − 4.7% (4.1%), respectively. Referenced to white matter, 4- and 8-month changes were − 0.33 (0.041) and − 0.015 (0.027) or − 3.5% (4.1%) and − 1.7% (2.8%), relative to whole cerebellum − 0.249 (0.148) and − 0.160 (0.102) or − 13.6% (8.6) and − 9.2% (6.32%), respectively, and referenced to bilateral parietal cortex − 2.8% (2.7%) and − 1.0% (2.2%). A comparison of left to right large parietal ROI (of which the implant and opposite side ROIs were subsets) showed a decrease of − 0.33 (0.041) and − 0.015 (0.027) or − 3.5% (4.1%) and − 1.7% (2.85).

In all cases, a decrease was observed at 4 months that partially but not completely was reduced in magnitude at 8 months (the period during which no sonications were performed). Wilcoxon signed-rank test results comparing baseline to 4 months resulted in *p*-values ranging from trend level (0.1, reference adjacent tissue and whole cerebellum) to not significant (other reference regions). For the participant with a sub-threshold implant ROI SUVR (Patient 10), changes ranged from 3% (referenced to adjacent tissue, white tissue, and bilateral parietal) to 9% (opposite hemisphere) at 4 months and from − 4 to + 4% at 8 months.

### FDG PET results

**Fig. 5:**
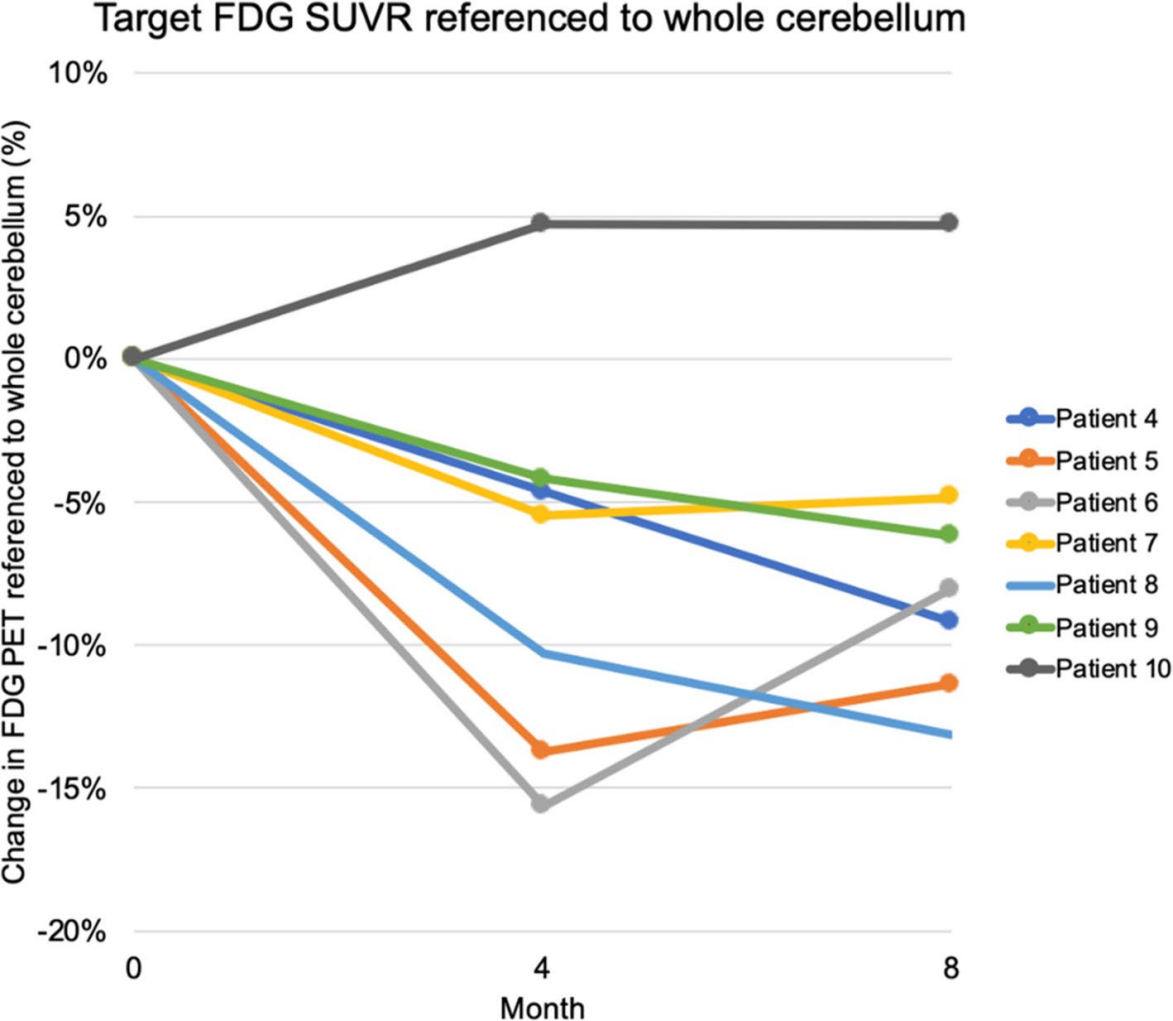
FDG PET changes in the target ROI (ROI1) referenced to the whole cerebellum. Overall, a blunting or plateau of decline was observed in the target region referenced to the whole cerebellum after 4 months

The participants exhibited decreases in the regional glucose metabolism, referenced to the whole cerebellum, in regions consistent with the overall pattern of metabolic decline. A blunting or plateau in decline was observed in the target region referenced to the whole cerebellum after 4 months (Fig. 5). When the target region or the broader left supramarginal region was compared to the right supramarginal region, a non-significant blunting of decline was also observed after 4 months.

### Results of the global-scale analyses and comparison with an external control cohort

Potential effects of BBB disruption further away from the device, i.e., within the whole cortex and a temporoparietal ROI, were also studied for both amyloid and FDG PET. The amyloid uptake remained on average stable between M0 and M8 (no statistical difference according to the Wilcoxon signed-rank test), while the FDG uptake decreased on average between M0 and M8 (significant difference in the left cortex and in both the left and right temporoparietal ROIs according to the Wilcoxon signed-rank test after correction for multiple comparisons following the Benjamini-Hochberg procedure) (see Additional file 1: Table S2).

No statistically significant difference at baseline or in the evolution between the subjects that received sonications, and the external control group (ADNI) was identified on amyloid, FDG PET, nor on cognition (Additional file 1: Fig. S2 & Table S3).

In the BBB disruption efficacy analysis, post-sonication Gd-DOTA concentration maps were computed from MRI T1 maps, and a sonicated ROI was compared with a non-sonicated control ROI. Detectable ultrasound-mediated BBB disruption was observed for 10/16 (62.5%) of the sessions with available T1 maps (Additional file 1: Table S1). Six of the nine patients (70%) had detectable BBB disruption for at least one of the two sessions with MR imaging. In the sessions with detected BBB disruption, the average brain Gd-DOTA enhancement volume difference between ROI and control was 0.81 ± 0.38 mL, and the average difference in Gd-DOTA quantity was 25.7 ± 8.5 μg. No significant difference or trend was found when comparing the two sessions with MRI data available (session 1 and session 3) for the same patient.

### Cognitive change results

No statistically significant change was observed on cognitive measures compared either to individual data or to cognitive change evidenced in the matched sample of ADNI participants (Additional file 1: Fig. S2).

## Discussion

In this study, the feasibility of repeated BBB disruption in early AD patients using an implantable ultrasound device was shown. A total of seven repeated sonication sessions, performed every 2 weeks, were performed in nine patients. The procedure’s tolerance had already been demonstrated in a group of nineteen patients with recurrent glioblastoma that received 65 sonication sessions prior to monthly infusion of carboplatin chemotherapy (median age = 59 years old) [50]. Our study confirms the safety of the device and sonication sessions to disrupt the BBB in a group of older (median age = 71 years old) and cognitively impaired individuals. One participant had a delirium that lasted for 2 h which occurred 2 days after a sonication. It was associated with the re-bleeding of a microbleed which was already present prior to study enrollment. This is reminiscent of an amyloid-related imaging anomaly of the hemorrhagic type (ARIAh) which is consistent with the trend we observed in amyloid clearance following the seven sonication sessions.

The SonoCloud-1 was implanted at the left parietotemporal junction centered on the left supramarginal gyrus. This tissue was selected because it was accessible (adjacent to the skull) for implant placement, is a site of typical AD pathology and decline in glucose metabolism, and is integral to the cognitive decline associated with AD. In particular, this region is an associative cerebral region involved in multiple functions including speech, calculation, and gestures, and an improvement in this area could have positive cognitive effects.

Although this was a pilot study, amyloid PET results suggest that SonoCloud-1 treatment is associated with a trend (not significant) of decreased amyloid burden in tissue proximal to the implant. This decrease was detected using multiple reference regions. These reductions were observed in subjects having a positive baseline amyloid burden in the target region. The lack of decreased signal in the subject with below-threshold amyloid in the target region is consistent with the signal having been associated with amyloid rather than a change in local blood flow or technical noise. No effect was observed further away from the region targeted by the implant.

The amyloid reductions observed in our study are consistent with several other recent studies that used focused ultrasound systems to treat Alzheimer’s patients. D’Haese et al. targeted the left or right hippocampus and performed three sonications every 2 weeks in combination with microbubbles in six patients to disrupt the BBB [28]. A slight reduction was observed between amyloid levels at inclusion and amyloid levels observed 7 days after the third (last) treatment, with a mean reduction of 5% across patients. No changes were observed in cognitive assessments. Park et al. targeted the bilateral frontal lobe and performed two sessions at 3-month intervals in five patients [51]. A mean volume of 21 cm^3^ was targeted, and BBB disruption was confirmed on MRI. A slight reduction in amyloid (− 1.6%) was observed at 3 months after the 2nd sonication along with a transient improvement in neuropsychiatric symptoms.

In another study using focused ultrasound in Alzheimer’s patients, Jeong et al. performed sonications using an external focused ultrasound system with microbubble infusions, targeting the right hippocampus, in four patients at ultrasound intensities below the threshold for visible BBB opening on MRI [52]. Glucose metabolism was increased in the superior frontal gyrus, middle cingulate gyrus, and fusiform gyrus at 2 weeks after sonication, and patients demonstrated mild improvement in measures of memory, executive, and global cognitive functions.

In our study, no cognitive effects of sonication were observed, but the small study sample size of nine patients, limited region of sonication, spatial location, and short follow-up may explain why no variation in cognitive assessments could be demonstrated. Targeting a larger region in the frontal cortex or the hippocampus may be better suited to showing cognitive improvements, as was done in the studies cited above [51, 52].

Recent large trial data from anti-amyloid immunotherapies indicates that cognitive improvement likely requires more complete removal of amyloid over a longer period of time [9, 53]. BBB opening using ultrasound coupled with these new antibody treatments could act synergistically and could be explored in further studies. The observation of cognitive improvement over shorter durations in the mouse model [15] may be attributable to the whole hemisphere/brain sonications that were able to be performed, coupled with a more rapid disease or response process.

### Limitations

Limitations of the present work included the limited sample size, study follow-up, and the spatially limited application of sonication. Longer trials may be needed to determine the frequency and duration of sonication required to maintain the effect. In addition, other than referencing external studies such as ADNI for comparison of amyloid accumulation rates, there were no controls for the study. As the first study on impaired aged patients, a small sample size was ethically acceptable, and given the need for invasive implant placement, it would not have been feasible to have a control group implanted with the device and given sham sonications. Despite these limitations, the ability to compare to control regions within subject provided a basis for preliminary findings regarding the effects upon amyloid. The volume of the implant ROI was relatively small and the portion and morphology of gray matter variable, increasing the opportunity for motion-induced noise.

## Conclusions

In summary, our results confirm that repeatedly opening the BBB in mild AD patients is both feasible and well-tolerated and may be associated with a reduction of amyloid burden. Recent pre-clinical studies also show that ultrasound alone (without microbubbles/BBB disruption) may have positive effects on cognition that we did not evidence in our trial, perhaps due to the sample size [54]. These findings as well as the potential for enhanced therapeutic bioavailability in the brain of this approach merit further studies of this new treatment approach for AD and other neurodegenerative diseases.

## Supplementary Information

**Additional file 1. **Supplementary analyses at the global scale. **Fig. S1**. Baseline amyloid load for each participant in regional SUVRs and in target (implant) region (ROI1). All participants had a positive amyloid load in the region targeted by the sonication except for Patient 10 (threshold of 1.1 assumed for amyloid positivity). **Fig. S2**. Violin plots representing the distribution of the annualized percent changes (APC) in PET uptake for the current study ("BOREAL", *n=*9) and reference cohort ("ADNI", *n*=45) populations. The APC were computed for both the cognitive (left) amyloid PET (middle) and FDG PET (right) in the large (top) and small (bottom) regions of interest for PET imaging. No statistical difference was observed between BOREAL and ADNI (Kruskal-Wallis H-test with the Benjamini-Hochberg procedure to correct for multiple testing). **Fig. S3**. Typical Gd-DOTA concentration map obtained after a successful sonication session. Images are reoriented in order to contain the central axis of the implanted ultrasound implant (shown in pink). The implant region of interest covering brain tissues in the white and gray matter targeted by the ultrasound beam is shown in green. A non-sonicated control region defined in the contralateral hemisphere is also shown. **Fig. S4**. Acoustic field simulated in the brain for the SonoCloud-1 device at a nominal pressure of 1.03 MPa. The nominal pressure is calibrated in water at the natural focus (red cross) during manufacturing. The acoustic field in brain is evaluated from the measurement in water and considering an attenuation of 0.6 dB/cm/MHz. **Table S1**. Evaluation of BBB-disruption efficacy with metrics computed from T1 maps acquired after sonication. The differences of Gd-DOTA marked with * are above the criterion for detectable BBB disruption (2 times the standard deviation of all control ROIs, i.e. 13.9 μg). **Table S2**. Regional standardized uptake value ratios (SUVR) obtained for the amyloid and FDG PET tracers at M0, M4 and M8, presented as average ± standard deviation. The amyloid uptake remained on average stable between M0 and M8 (no statistical difference according to the Wilcoxon signed rank test), while the FDG uptake decreased on average between M0 and M8 (significant difference in the left cortex, and in both the left and right angular + supramarginal + superior temporal gyri according to the Wilcoxon signed rank test after correction for multiple comparisons following the Benjamini-Hochberg procedure). L: left, R: right. **Table S3**. Comparison of the BOREAL and ADNI populations. No statistical difference exists in terms of age and mini mental state examination (MMSE) score (Kruskal-Wallis H-test) between the BOREAL and ADNI populations, nor in terms of regional PET standardized uptake value ratio (SUVR) at baseline and annualized percent change (APC) computed in both the large and small regions of interest (Kruskal-Wallis H-test with the Benjamini-Hochberg procedure to correct for multiple testing). L: left, R: right.

## Data Availability

Data is available upon reasonable request to the corresponding author.

## Abbreviations

AD: Alzheimer’s disease
ADNI: Alzheimer’s Disease Neuroimaging Initiative
AE: Adverse event
BBB: Blood-brain barrier
CDR-SB: Clinical Dementia Rating Scale Sum of Boxes Score
CSF: Cerebrospinal fluid
CTCAE: Common terminology criteria for adverse events
FAB: Frontal Assessment Battery
FCSRT: Free and Cued Selected Reminding Test
FDG: Fludeoxyglucose
GBM: Glioblastoma
Gd: Gadolinium
MADRS: Montgomery-Åsberg Depression Rating Scale
MCI: Mild cognitive impairment
MMSE: Mini-Mental State Examination
MPa: Megapascal
MRI: Magnetic resonance imaging
PET: Positron emission tomography
rGBM: Recurrent glioblastoma
ROI: Region of interest
SPM: Statistical parametric mapping
STAI: State-Trait Anxiety Inventory
SUV: Standard uptake value
TMT: Trail Making Test
US: Ultrasound
US-BBBD: Ultrasound-induced blood-brain barrier disruption
