# Supplementary material for: Pilot study of repeated blood-brain barrier disruption in patients with mild Alzheimer’s disease with an implantable ultrasound device
Source: Alzheimers Res Ther. 2022 Mar 8;14:40. doi: 10.1186/s13195-022-00981-1 (PMC8905724; doi:10.1186/s13195-022-00981-1)
Supplement: Supplementary file 1 — Additional file 1. Supplementary analyses at the global scale. Fig. S1. Baseline amyloid load for each participant in regional SUVRs and in target (implant) region (ROI1). All participants had a positive amyloid load in the region targeted by the sonication except for Patient 10 (threshold of 1.1 assumed for amyloid positivity). Fig. S2. Violin plots representing the distribution of the annualized percent changes (APC) in PET uptake for the current study ("BOREAL", n=9) and reference cohort ("ADNI", n=45) populations. The APC were computed for both the cognitive (left) amyloid PET (middle) and FDG PET (right) in the large (top) and small (bottom) regions of interest for PET imaging. No statistical difference was observed between BOREAL and ADNI (Kruskal-Wallis H-test with the Benjamini-Hochberg procedure to correct for multiple testing). Fig. S3. Typical Gd-DOTA concentration map obtained after a successful sonication session. Images are reoriented in order to contain the central axis of the implanted ultrasound implant (shown in pink). The implant region of interest covering brain tissues in the white and gray matter targeted by the ultrasound beam is shown in green. A non-sonicated control region defined in the contralateral hemisphere is also shown. Fig. S4. Acoustic field simulated in the brain for the SonoCloud-1 device at a nominal pressure of 1.03 MPa. The nominal pressure is calibrated in water at the natural focus (red cross) during manufacturing. The acoustic field in brain is evaluated from the measurement in water and considering an attenuation of 0.6 dB/cm/MHz. Table S1. Evaluation of BBB-disruption efficacy with metrics computed from T1 maps acquired after sonication. The differences of Gd-DOTA marked with * are above the criterion for detectable BBB disruption (2 times the standard deviation of all control ROIs, i.e. 13.9 μg). Table S2. Regional standardized uptake value ratios (SUVR) obtained for the amyloid and FDG PET tracers at M0, M4 and M8, pres [file 13195_2022_981_MOESM1_ESM.docx]

# SUPPLEMENTARY MATERIALS

**Supplementary analyses at the global scale**

Potential effects upon amyloid and FDG PET were additionally evaluated by comparing SUVRs for whole cortex and a temporoparietal ROI in the hemisphere of the implant versus the opposite hemisphere and a slightly different set of processing steps. The T1w MR images acquired during both the FDG and amyloid PET/MR acquisitions at M0, M4 and M8 were corrected for intensity non-uniformity following a non-parametric intensity non-uniformity normalization method (50) and each PET image was rigidly registered to its corresponding, simultaneously acquired, T1w MR image (51). The T1w MR and PET images were then globally aligned in a common space via an affine groupwise registration (51). The T1w MR images were further parcellated into 143 different regions using a multi-atlas propagation and fusion algorithm (52). A consensus parcellation was obtained by computing the intersection of the individual parcellations. The PET images were intensity-normalized using the average uptake in reference regions that were extracted from the consensus parcellation to obtain standardized uptake value ratio (SUVR) maps. As we analyze longitudinal data, a region comprising the cerebellar lobules III, IV, V, VI and entire vermis was used for the FDG PET images (53) and a region comprising the eroded white matter, whole cerebellum, and brainstem was used for the amyloid PET images (54). Two ROIs, also extracted from the consensus parcellation, were considered when analyzing the PET images: a large region corresponding to the whole cortex and a small region surrounding the device comprising the angular, supramarginal and superior temporal gyri. For both the whole cortex and temporoparietal ROIs, each hemisphere was analyzed separately. To study the evolution of the PET SUVRs between M0 and M8, we computed the annualized percent change (APC) as:

$$APC= \left[ \left( \frac{{PET SUVR}_{M8}}{{PET SUVR}_{M0}} \right)^{\frac{1}{M8-M0}}-1 \right]\times100$$

with the time difference between M0 and M8 expressed in years.

**Supplementary Figure 1.** Baseline amyloid load for each participant in regional SUVRs and in target (implant) region (ROI1). All participants had a positive amyloid load in the region targeted by the sonication except for Patient 10 (threshold of 1.1 assumed for amyloid positivity).

**
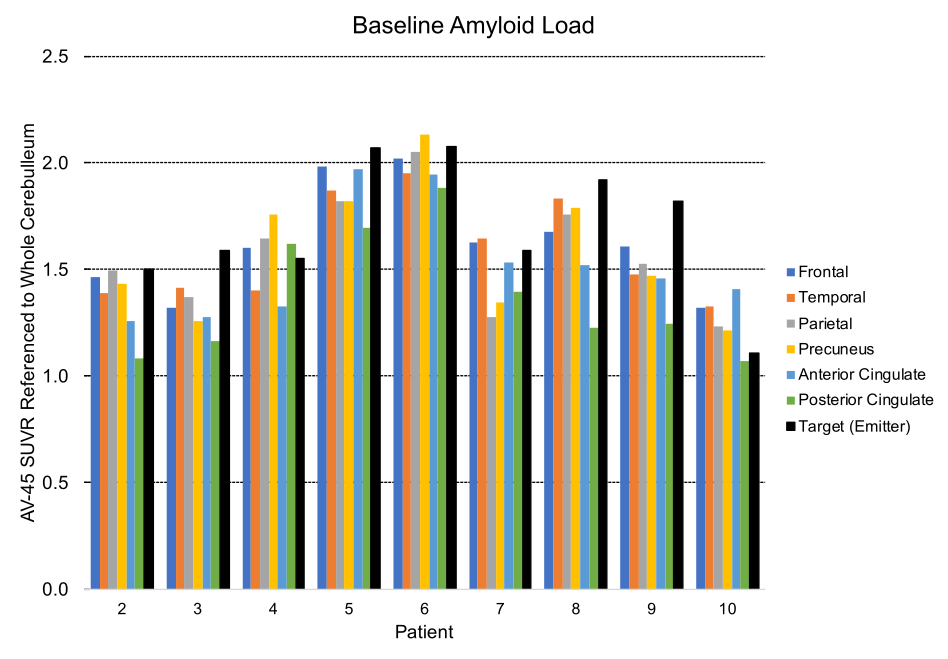
**

**Supplementary Figure 2.** Violin plots representing the distribution of the annualized percent changes (APC) in PET uptake for the current study ("BOREAL", n=9) and reference cohort ("ADNI", n=45) populations. The APC were computed for both the cognitive (left) amyloid PET (middle) and FDG PET (right) in the large (top) and small (bottom) regions of interest for PET imaging. No statistical difference was observed between BOREAL and ADNI (Kruskal-Wallis H-test with the Benjamini-Hochberg procedure to correct for multiple testing).


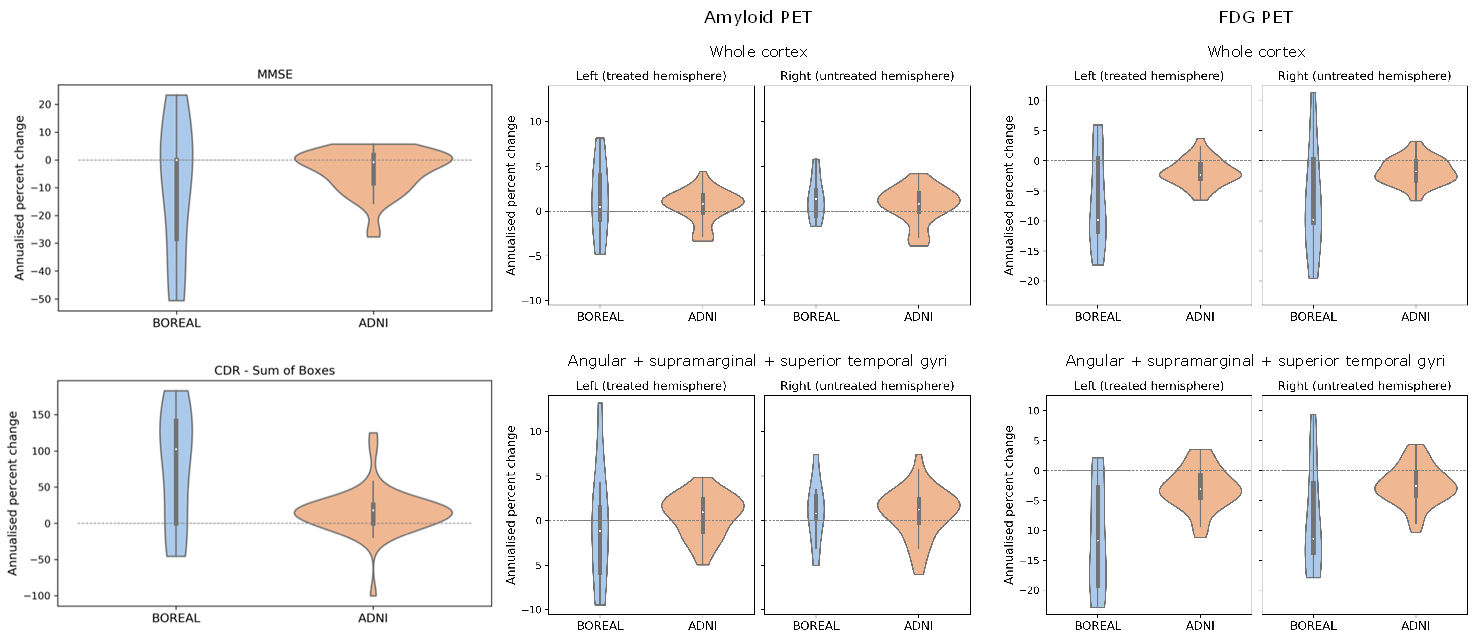


**Supplementary Figure 3.** Typical Gd-DOTA concentration map obtained after a successful sonication session. Images are reoriented in order to contain the central axis of the implanted ultrasound implant (shown in pink). The implant region of interest covering brain tissues in the white and gray matter targeted by the ultrasound beam is shown in green. A non-sonicated control region defined in the contralateral hemisphere is also shown.


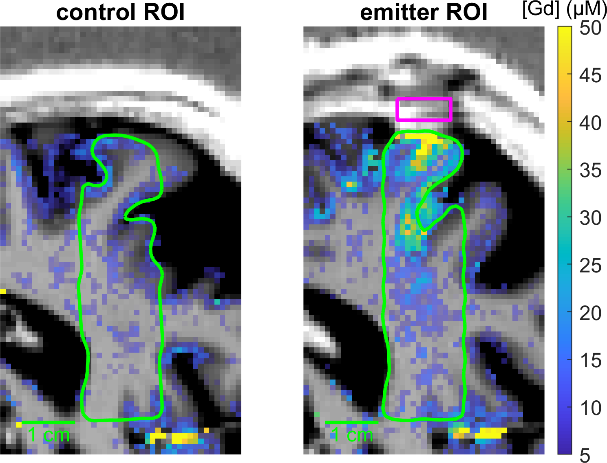


**Supplementary Figure 4.** Acoustic field simulated in the brain for the SonoCloud-1 device at a nominal pressure of 1.03 MPa. The nominal pressure is calibrated in water at the natural focus (red cross) during manufacturing. The acoustic field in brain is evaluated from the measurement in water and considering an attenuation of 0.6 dB/cm/MHz.

**
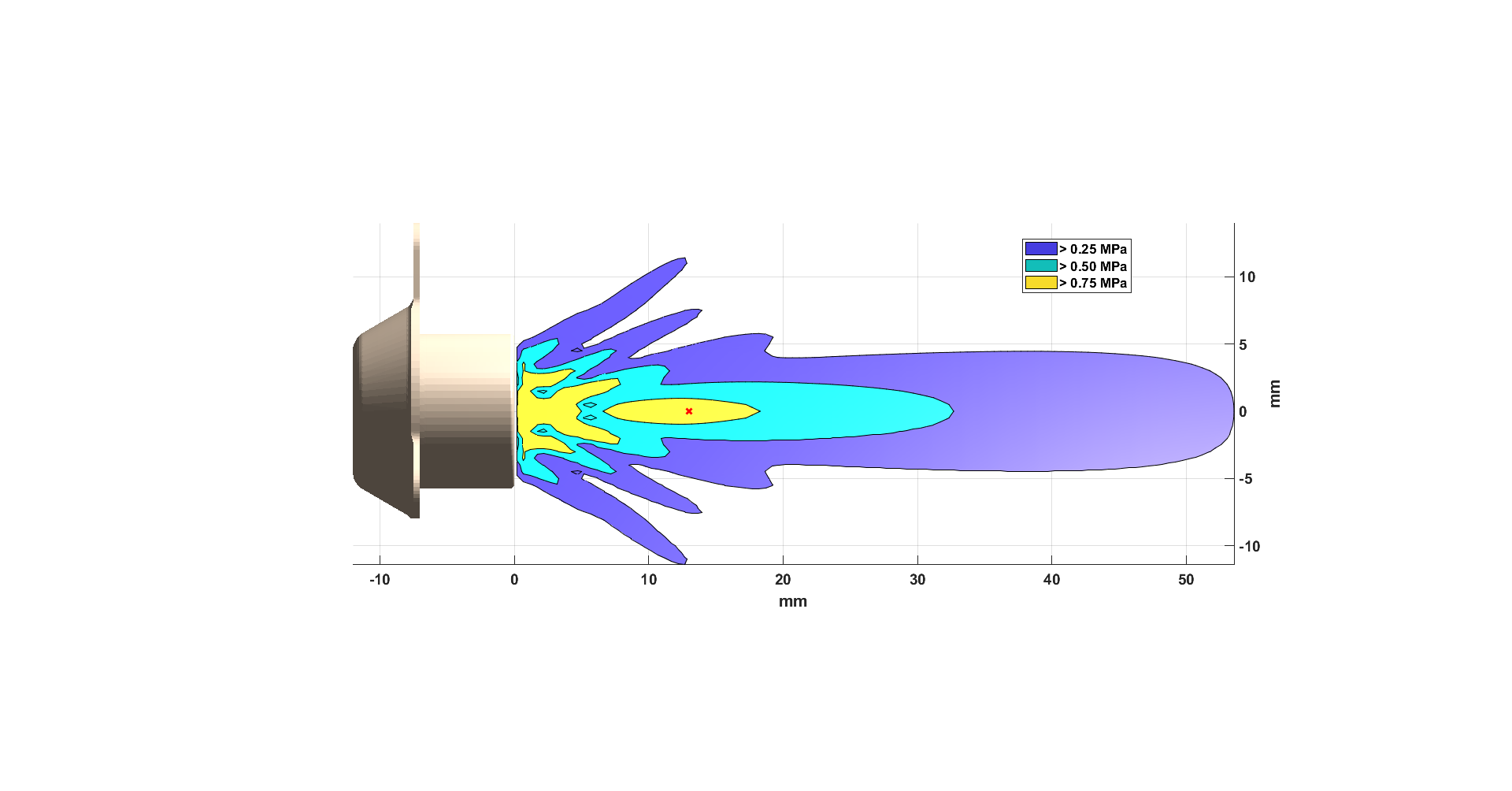
**

**Supplementary Table 1.** Evaluation of BBB-disruption efficacy with metrics computed from T1 maps acquired after sonication. The differences of Gd-DOTA marked with * are above the criterion for detectable BBB disruption (2 times the standard deviation of all control ROIs, i.e. 13.9 µg)

| Patient | Session | BBB opening on visual inspection | Gd-DOTA ROI  (µg) | Gd-DOTA control (µg) | Difference | Enhanced volume ROI  (mL) | Enhanced volume control (mL) | Difference |
| --- | --- | --- | --- | --- | --- | --- | --- | --- |
| 2 | 1 | 2 | 30.2 | 13.8 | 16.4* | 0.90 | 0.29 | 0.61 |
| 2 | 3 | 2 | 42.4 | 17.7 | 24.7* | 1.22 | 0.29 | 0.93 |
| 3 | 1 | 2 | 54.7 | 16.7 | 37.9* | 1.93 | 0.42 | 1.51 |
| 3 | 3 | 2 | 51.9 | 25.2 | 26.7* | 1.06 | 0.42 | 0.64 |
| 4 | 1 | 2 | 15.8 | 10.8 | 5.1 | 0.57 | 0.33 | 0.25 |
| 4 | 3 | 1 | 19.6 | 15.8 | 3.8 | 0.63 | 0.32 | 0.31 |
| 5 | 1 | 3 | 54.0 | 12.3 | 41.6* | 1.59 | 0.25 | 1.34 |
| 5 | 3 | 2 | 31.2 | 12.1 | 19.1* | 0.59 | 0.26 | 0.33 |
| 6 | 3 | 2 | 4.0 | -0.2 | 4.3 | 0.32 | 0.30 | 0.02 |
| 7 | 1 | 0 | 9.9 | 6.2 | 3.7 | 0.35 | 0.28 | 0.08 |
| 7 | 3 | 0 | 15.7 | 12.6 | 3.2 | 0.45 | 0.28 | 0.17 |
| 8 | 1 | 1 | 46.6 | 25.6 | 21.0* | 0.83 | 0.32 | 0.51 |
| 8 | 3 | 1 | 22.4 | 18.1 | 4.3 | 0.52 | 0.32 | 0.20 |
| 9 | 3 | 3 | 35.8 | 7.6 | 28.2* | 1.11 | 0.33 | 0.77 |
| 10 | 1 | 2 | 31.6 | 6.4 | 25.2* | 1.25 | 0.27 | 0.98 |
| 10 | 3 | 2 | 22.4 | 6.4 | 16.1* | 0.78 | 0.27 | 0.51 |

**Supplementary Table 2**. Regional standardized uptake value ratios (SUVR) obtained for the amyloid and FDG PET tracers at M0, M4 and M8, presented as average ± standard deviation. The amyloid uptake remained on average stable between M0 and M8 (no statistical difference according to the Wilcoxon signed rank test), while the FDG uptake decreased on average between M0 and M8 (significant difference in the left cortex, and in both the left and right angular + supramarginal + superior temporal gyri according to the Wilcoxon signed rank test after correction for multiple comparisons following the Benjamini-Hochberg procedure). L: left, R: right.

|  | |  |  |  | SUVR | | | | | | | | | Evolution between M0 and M8 | |
| --- | --- | --- | --- | --- | --- | --- | --- | --- | --- | --- | --- | --- | --- | --- | --- |
|  | |  |  |  | M0 | | | M4 | | | M8 | | | p values | Corrected  p values |
| Amyloid  PET | Whole cortex | | L | | 0.73 | ± | 0.23 | 0.74 | ± | 0.23 | 0.73 | ± | 0.23 | 0.413 | 0.551 |
|  |  |  | R | | 0.73 | ± | 0.23 | 0.76 | ± | 0.24 | 0.74 | ± | 0.23 | 0.206 | 0.551 |
|  | Angular + supramarginal + superior temporal gyri | | L | | 0.77 | ± | 0.24 | 0.78 | ± | 0.24 | 0.76 | ± | 0.24 | 0.831 | 0.831 |
|  |  |  | R | | 0.76 | ± | 0.24 | 0.80 | ± | 0.25 | 0.76 | ± | 0.24 | 0.413 | 0.551 |
| FDG PET | Whole cortex | | L | | 0.97 | ± | 0.30 | 0.93 | ± | 0.29 | 0.92 | ± | 0.29 | 0.032 | **0.043** |
|  |  |  | R | | 0.97 | ± | 0.30 | 0.96 | ± | 0.30 | 0.92 | ± | 0.29 | 0.067 | 0.067 |
|  | Angular + supramarginal + superior temporal gyri | | L | | 0.92 | ± | 0.29 | 0.85 | ± | 0.27 | 0.84 | ± | 0.27 | 0.014 | **0.043** |
|  |  |  | R | | 0.92 | ± | 0.29 | 0.91 | ± | 0.28 | 0.87 | ± | 0.27 | 0.024 | **0.043** |

**Supplementary Table 3.** Comparison of the BOREAL and ADNI populations. No statistical difference exists in terms of age and mini mental state examination (MMSE) score (Kruskal-Wallis H-test) between the BOREAL and ADNI populations, nor in terms of regional PET standardized uptake value ratio (SUVR) at baseline and annualized percent change (APC) computed in both the large and small regions of interest (Kruskal-Wallis H-test with the Benjamini-Hochberg procedure to correct for multiple testing). L: left, R: right.

|  | |  | |  |  | BOREAL | | | ADNI | | | p values | Corrected p values |
| --- | --- | --- | --- | --- | --- | --- | --- | --- | --- | --- | --- | --- | --- |
| Number of participants | | | | | | 9 | | | 45 | | | - | - |
| Number of female participants | | | | | | 5 | | | 25 | | | - | - |
| Age at baseline | | | | | | 71.22 | ± | 4.15 | 72.31 | ± | 5.67 | 0.659 | **-** |
| MMSE at baseline | | | | | | 23.78 | ± | 2.68 | 24.58 | ± | 2.60 | 0.319 | **-** |
| Amyloid  PET | Whole cortex | | L | SUVR | | 0.79 | ± | 0.09 | 0.78 | ± | 0.12 | 0.880 | 0.880 |
|  |  |  |  | APC | | 1.23 | ± | 4.34 | 0.54 | ± | 1.82 | 0.862 | 0.954 |
|  |  |  | R | SUVR | | 0.79 | ± | 0.10 | 0.77 | ± | 0.12 | 0.702 | 0.880 |
|  |  |  |  | APC | | 1.42 | ± | 2.46 | 0.67 | ± | 2.01 | 0.570 | 0.954 |
|  | Angular + supramarginal + superior temporal gyri | | L | SUVR | | 0.84 | ± | 0.08 | 0.80 | ± | 0.14 | 0.618 | 0.880 |
|  |  |  |  | APC | | -0.67 | ± | 6.95 | 0.57 | ± | 2.34 | 0.372 | 0.954 |
|  |  |  | R | SUVR | | 0.83 | ± | 0.10 | 0.79 | ± | 0.14 | 0.602 | 0.880 |
|  |  |  |  | APC | | 0.95 | ± | 3.62 | 0.87 | ± | 2.75 | 0.954 | 0.954 |
| FDG PET | Whole cortex | | L | SUVR | | 1.05 | ± | 0.10 | 1.06 | ± | 0.10 | 0.898 | 0.898 |
|  |  |  |  | APC | | -6.50 | ± | 8.26 | -1.93 | ± | 2.21 | 0.092 | 0.092 |
|  |  |  | R | SUVR | | 1.06 | ± | 0.08 | 1.06 | ± | 0.10 | 0.898 | 0.898 |
|  |  |  |  | APC | | -6.65 | ± | 9.36 | -1.76 | ± | 2.18 | 0.059 | 0.078 |
|  | Angular + supramarginal + superior temporal gyri | | L | SUVR | | 0.99 | ± | 0.16 | 1.02 | ± | 0.15 | 0.602 | 0.898 |
|  |  |  |  | APC | | -11.21 | ± | 9.61 | -3.00 | ± | 3.47 | 0.032 | 0.076 |
|  |  |  | R | SUVR | | 1.00 | ± | 0.12 | 1.03 | ± | 0.15 | 0.494 | 0.898 |
|  |  |  |  | APC | | -8.33 | ± | 9.36 | -2.48 | ± | 3.25 | 0.038 | 0.076 |
